# Genotoxicity and cytotoxicity of sevoflurane in two human cell lines in vitro with ionizing radiation

**Published:** 2014-09-30

**Authors:** Miguel Alcaraz, Samuel Quesada, David Armero, Rocio Martin-Gíl, Amparo Olivares, Daniel Achel

**Affiliations:** 1 Radiology and Physical Medicine Department. Faculty of Medicine/Dentistry. University of Murcia. 30100 Espinardo. Murcia. Spain; 2 Nursing Department. Faculty of Nursing. University of Murcia. 30100 Espinardo. Murcia. Spain; 3 Anesthesiology Department. Virgen de la Arrixaca University Hospital, 30120. El Palmar. Murcia. Spain; 4 Applied Radiation Biology Centre, Radiological and Medical Sciences Research Institute, Ghana Atomic Energy Commission, Legon-Accra, Ghana

**Keywords:** Anaesthesia, genotoxicity, micronucleus, radiation effects, sevofluorane

## Abstract

**Objective:**

To determine the *in vitro* toxicity of different concentrations of sevoflurane in cells exposed to X-ray.

**Methods:**

The genotoxic effects of sevofluorane were studied by means of the micronucleus test in cytokinesis-blocked cells of irradiated human lymphocytes. Subsequently, its cytotoxic effects on PNT2 (normal prostate) cells was determined using the cell viability test (MTT) and compared with those induced by different doses of X-rays.

**Results:**

A dose- and time-dependent cytotoxic effect of sevofluorane on PNT2 cells was determined (*p *>0.001) and a dose-dependent genotoxic effect of sevofluorane was established (*p *>0.001). Hovewer, at volumes lower than 30 μL of sevofluorane at 100%, a non-toxic effect on PNT2 cells was shown.

**Conclusion:**

Sevofluorane demonstrates a genotoxic capacity as determined *in vitro* by micronucleus test in cytokinesis-blocked cells of irradiated human lymphocytes.

## Abstract (es)

**Objetivo:**

Determinar la capacidad genotóxica del anestésico sevofluorano en en células expuestas a radiación ionizante.

**Métodos:**

**L**a genotoxicidad del sevofluorane se determinó mediante el test del bloqueo citocinético de linfocitos humanos irradiados bloqueados con citochalasina. La capacidad citotóxica se determino mediante el test de viabilidad celular e inhibición del crecimiento celular (MTT) en células PNT2 (epiteliales de próstata), comparando sus resultados con los inducidos por diferentes dosis de rayos X.

**Resultados:**

Se ha determinado un efecto citotóxico del sevofluorane sobre las células PNT2 que presenta correlación con la dosis administrada y el tiempo de estudio utilizado (*p *>0.001), así como un efecto genotóxico con características dosis-dependientes *(p *>0.001). Sin embargo, con volúmenes de sevofluorane puro inferiores a 30 μL no encontramos efecto citotóxico sobre las células PNT2.

**Conclusión:**

Sevofluorane muestra una significativa capacidad genotóxica *in vitro* determinada mediante el test de micronúcleos en linfocitos humanos irradiados con bloqueados citocinético mediante citochalsina.

## Introduction 

Sevoflurane is a widely used general anaesthetic especially suitable in short surgical procedures and ambulatory surgery 1. Its main advantages are the quick induction of anaesthesia while maintaining spontaneous breathing and contribution to hemodynamic stability of the patient 2. Its toxicity was discovered while in the pursuit of the effect of sevofluorane on hepatic function 3. It has been implicated in the production of toxic metabolites, and the induction malignant hyperthermia 4.

Exposure to inhalation anaesthetics generates "triggerings" of small quantities of reactive oxygen species (ROS), either directly, by interacting with the mitochondrial electron transport chain, or indirectly, through a signalling cascade in which G-protein-coupled receptors, proteinkinases, and mitochondrial ATP-sensitive potassium (K_ATP_) channels play important roles. This attenuation of respiration may cause leakage of electrons from the inner mitochondrial matrix and augment ROS generation 5 ^,^ 6. Sevofluorane can also directly trigger the formation of peroxynitrite and significantly increase intracellular H_2_O_2_ and/or peroxide, superoxide, and nitric oxide (NO) in peripheral polymorphonuclear neutrophils after 1 h of treatment. Furthermore, the intensification of intracellular glutathione (GSH) depletion in neutrophils has been demonstrated. These results are important for demonstrating oxidative stress induced by administration of sevofluorane by means of increasing the concentration of ROS 6 ^,^ 7.

Oxidative stress induced by increasing levels of ROS is the postulated mechanism by which genotoxic damage is induced by ionizing radiations. The micronucleus test has been successfully used to assay for both *in vivo* and *in vitro* effects of this type of genotoxic damage 8 ^,^ 9. Using this assay technique, the administration of diverse antioxidant substances have shown genoprotective effects against chromosomal damage induced by the ionizing radiations 10 ^-^ 12.

In this study we attempt to determine the possible genotoxic effect of sevoflurane using the micronuclei test. To do this, we will quantify the number of micronuclei per 1,000 binucleated cells in blood samples exposed to sevofluoraneo and compare it with blood samples from controls and blood samples exposed to ionizing radiation, whose genotoxic effect has been shown by various authors.

## Materials and Methods

### Chemicals and reagents

Sevofluorane was obtained from Abbot (Madrid, Spain) and was administered pure in different volumes (20-40 µL). RPMI 1640, F10, PHA, DMSO, cytochalasin B, streptomycin, penicillin, phosphate buffered saline (PBS) and 3-(4,5-dimethyl-2-thiazolyl)-2,5-diphenyl-2h-tetrazolium bromide (MTT), were obtained from Sigma-Aldrich Química S.A (Madrid, Spain). Foetal bovine serum was obtained from Gibco (USA); glacial acetic acid and ethanol were obtained from Scharlao SL (Madrid, Spain), methanol was obtained from Panreac (Madrid, Spain); 5% sodium heparin was obtained from Laboratorios Rovi (Madrid, Spain) and 95% Rosmarinic acid (RO) was obtained from Extrasynthese (Genay, France).

### Cell survival curve, viability quantification and MTT test

### Cell line and culture conditions

The PNT2 cell line used was obtained from the European Collection of Cell Cultures (ECACC) Health Protection Agency Culture Collection (Catalogue nº 95012613, HPACC, UK). Tests were carried out to confirm the absence of *Mycoplasma* spp. throughout the study. The PNT2 cells were cultured in RPMI 1640 supplemented with foetal bovine serum (FBS) (10%), glutamine (2 mM) and streptomycin plus penicillin (100 µg/mL and 100 IU/mL, respectively). All the processes were carried out in a Cultair ASB type II vertical laminar flow chamber. The PNT2 cultures were kept at 37° C and 95% relative humidity, in 5% CO_2 _atmosphere, in a Cytoperm incubator. The culture medium was changed every 2 days or when acidification was indicated by the pH indicator (phenol red). After irradiation, all microplates were incubated for an additional 24, 48 and 72 h, and no medium changes were performed. To determine the possible radioprotective effects we included positive control wells containing 20 μL of DMSO (0.2%) and 25 μM RO to the cell survival studies.

### MTT test

To analyze the effects of sevofluorane on cell viability and PNT2 cell survival, we used the 3-(4,5-dimethylthiazol-2-yl)-2,5-diphenyl tetrazolium bromide (MTT) assay for 24 or 48 h.

Briefly, the cell cultures were incubated in 200 µL growth medium and allowed to adhere for 24 h. After treatment with the above mentioned incubation doses of sevofluorane, and for the mentioned times, supplemented growth medium and 50 µL of MTT (5 mg/mL) were added to each well in 96 well plates and the microplates were further incubated at 37° C for 4 h in a 5% CO_2_ atmosphere. Afterwards, the plates were centrifuged at 90 rpm for 8 min to carefully remove the medium and non-metabolized MTT, 100 µL of DMSO was added to each well to solubilize the MTT taken up by the living cells. After shaking for 30 min at room temperature, the plates were read with a Multiskan MCC/340P spectrophotometer using 570 nm for the reading and 690 nm for the reference wavelengths. The negative control wells were used for the baseline zero. Each experiment was repeated on three occasions.

### Genototoxic Effect: MN (MNCB)

Blood samples and irradiation procedure Human peripheral blood were drawn from six healthy young non-smoking female donors into heparinized tubes. Sevofluorane was administered at 100% at three different volumes (5, 20 and 40 μL); 20 μL RO (25 µM) and DMSO (0.2%) respectively were added to 2 mL of blood to determine their possible genoprotective effects and included as positive controls. Samples were homogenized just before X-irradiation.

### Culture technique

The micronucleus (MN) assay was carried out on the irradiated lymphocytes after X-irradiation, with the following cytokinesis-blocking (MNCB) method described by Fenech 13 and adapted by International Atomic Energy Agency (2011). Briefly: whole blood samples (0.5 mL) were cultured at 37° C for 72 h in 4.5 mL of F-10 medium containing 15% foetal bovine serum, 1.6 µg/mL of phytohaemaglutinin, 1% penicillin/streptomycin and, 1 µg/mL of glutamine. Forty-four hours after initiation of the lymphocyte cultures, 150 µL of cytochalasin B was added at a concentration of 6 µg/mL. At 72 h the lymphocytes were treated with hypotonic solution (KCl, 0.075 M) for 3 min and fixed using methanol: acetic acid (3:1). Air-dried slide preparations were made and stained with May-Grünwald Giemsa 24 h later. Each experiment was repeated on three occasions.

### Scoring of Micronucleus

Triplicate cultures were analysed for each volume of sevofluorane used. In each, at least 3,000 cytokinesis-blocked cells (CB cells) (MN/500 CB) were examined by two specialists using a Zeiss light microscope (Oberkochem, Germany) with 400x magnification to examine the slides and 1,000 X magnification to confirm the presence or absence of MN in the cells (3,000 CB/sample studied), according to recommended published criteria 8 ^,^ 9.

### Irradiation

The samples were exposed to X-rays with an Andrex SMART 200E instrument (YXLON International, Hamburg, Germany) operating at 4.5 mA, 36 cm FOD, at room temperature. The radiation doses were monitored by a UNIDOS^®^ Universal Dosimeter with PTW Farme^®^ ionization chambers TW 30010 (PTW-Freiburg, Freiburg, Germany) in the radiation cabin and the X-rays doses were confirmed by means of thermoluminescent dosimeters (TLDs) (GR-200, Conqueror Electronics Technology Co. Ltd, China). The TLDs were supplied and measured by CIEMAT (Ministry of Industry and Energy, Spain). In the micronucleus test with cytokinesis-blocked (CBMN) of human lymphocyte cells 2 Gy of irradiation was administered, whereas different doses of X-rays (5, 10, 15, 20 and 0 Gy as control) were used in the Cell survival Curve and viability quantification (MTT test).

### Statistical analysis

In the genotoxicity study, the degree of dependence and correlation between variables were assessed using analysis of variance complemented by a contrast of means (*p*< 0.05). Quantitative means were compared by regression and linear correlation analysis. In addition, we used the formula described by Sarma and Kesavan (1993) 8 ^,^ 9 to evaluate the Magnitude of Protection (%)= ((F_control_ _irradiated_-F_treated irradiated _)/F_control irradiated_)x100. Where F_control irradiated_= frequency of MN in untreated but irradiated blood lymphocytes and F_treated irradiated_= frequency of MN in blood lymphocytes treated with the substances and irradiated.

In the cytotoxicity assays, an analysis of variance (ANOVA) of repeated means was used to compare the percentages of surviving cells in the cultures with different concentrations of sevofluorane. This was complemented by least significant deference analyses to contrast pairs and means. The analyses were carried out by logarithmically transforming the data to comply with ANOVA conditions.

## Results

**Figure 1.**
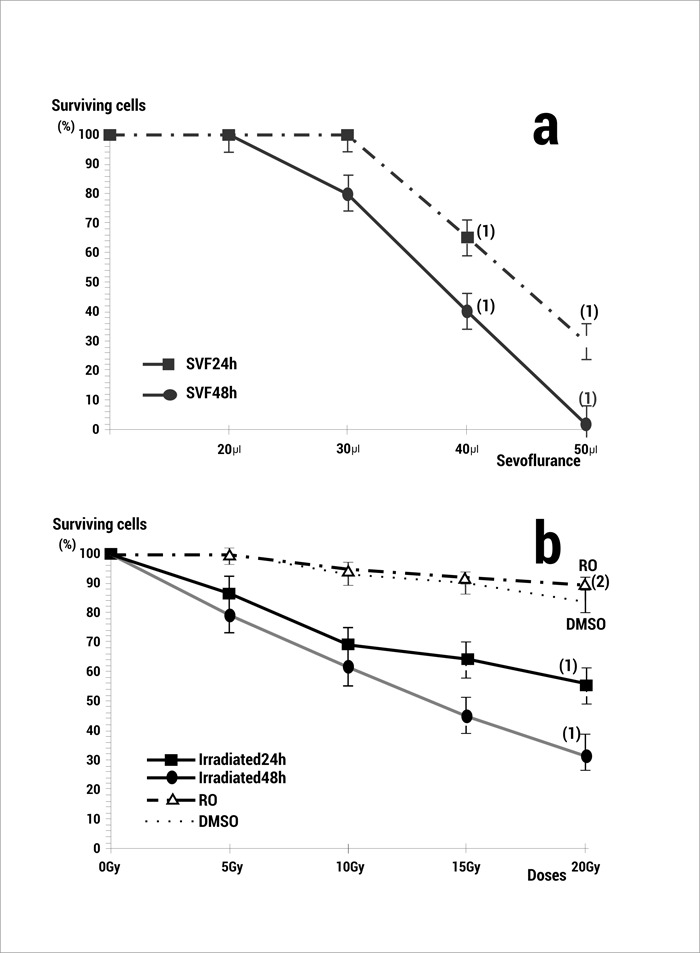
a) Effect of different volumes of sevofluorane on PNT2 cell viability. b) Radiation dose effects on PNT2 cell viability after 24 and 48 h incubation. Results are expressed as a percentage of surviving PNT2 cells in the control (RO: rosmarinic acid 25 μM irradiated and 48 h incubation; DMSO: Dimetyl sulphoxide 0.2% irradiated and incubated 48h) (1) *p *<0.001 versus control, (2) *p *<0.001 versus irradiated control.

In the cytotoxicity studies, the treatment of PNT2 cells with increasing volumes of sevofluorane for 24 and 48 h caused a dose- and time-dependent decrease in cell viability (*p *<0.001) (Fig. 1a). All the volumes in excess of 30 µL showed a significant degree of cytotoxicity (Fig. 1a). Radiation alone also caused a dose- and time-dependent decrease in cell viability (*p *<0.001) (Fig. 1b). Administration of 20 µL of RO (25 µM) or DMSO (0.2%) before the X-irradiation increased the survival of the PNT2 cells showing a significant radioprotective capacity (*p *<0.001) (Fig 1b).

**Figure 2.**
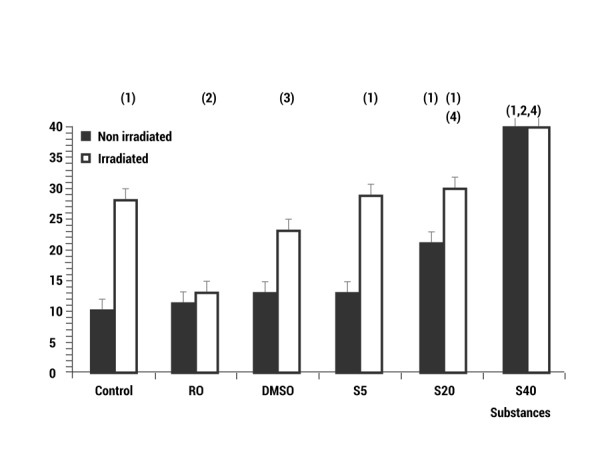
Genotoxic effect (Frequency of MN/500CB) of different volumes of sevofluorane and radiation: C control, RO Rosmarinic acid, DMSO Dimethyl sulfoxide and S_5_, S_20_, S_40_ different volumes of sevofluorane (µL) administered alone or before X-irradiation ((1) *p *<0.001 *versus* non-irradiated control, (2) *p *<0.001 *versus *irradiated control, (3) *p*< 0.01 *versus *irradiated control, (4) *p *<0.001 *versus* S_5_ non-irradiated.

In the genotoxic study, the basal frequency of the MN/500 CB was 10±2 MN/500 CB for the non-irradiated control of the human lymphocytes used in the cytome assay. Irradiation with 2 Gy of X-rays produced a significant increase in the appearance of MN, which reached 28±4 MN/500 CB (*p *<0.001), expressing a genotoxic damage induced by the X-rays (Fig. 2). The administration of RO and DMSO used as positive control of a radioprotective agent, led to a significant drop in the frequency of MN when administered before irradiation (*p *<0.001 and *p *<0.01, respectively). This expresses the genoprotective capacity of these substances against X-ray induced chromosome damage (Fig. 2), and demonstrate protection factors of 53.6% and 18.0% respectively. 

The administration of sevofluorane caused a dose-dependent increase in the frequency of MN compared with the controls (*p *<0.001) (Fig. 1b) signifying a genotoxic effect induced by sevofluorane. The genotoxic effects caused by the administration of sevofluorane does not show significant difference with respect to that caused by treatments with sevofluorane and irradiation except when much smaller (5 μL) doses of sevofluorane (*p *<0.01) are used.

## Discussion

The frequency of micronuclei is a reliable measure of both chromosome loss and breakage, making it unique compared to other cytogenetic tests. Chromosome damage is an indicator of genotoxicity and may ultimately result in aneuploid induction, and is also an important event in carcinogenesis 6 ^,^ 13. 

Our intention was to compare chromosomal damage induced by sevofluorane and X-rays by evaluating its genotoxic capacity. In previous studies, we determined the genotoxic capacity of ionizing irradiation *in vivo* with X-rays by the MN assay 14 ^,^ 15, *in vitro* with gamma irradiation 10 ^-^ 12 ^,^ 16 and at high irradiation doses^17^ or at the sensitivity threshold of the test (48 cGy) 18 ^,^ 19. We also used the MN test to determine the genoprotective effects of different antioxidant substances against chromosomic damage induced by X-rays *in vivo* on mouse bone marrow PCEs 14 ^,^ 15, or by gamma irradiation in lymphocyte cultures blocked with cytochalasin B 9 ^,^ 16 ^,^ 18 and in the presence or absence of different chemical protective substances with or without Sulphur containing compounds 10 ^,^ 14 ^,^ 15. 

The results obtained from these studies pointed to similar genotoxic capacities to the doses of X-rays used and a similar genoprotective capacities of the antioxidant substances assayed, especially when the antioxidants were present in the biochemical medium before irradiation *in vivo* on bone marrow of mice 10 ^,^ 14 and *in vitro* when human lymphocytes were kinetically blocked by cytochalasin-B 8 ^,^ 9.

Our studies show the dose-dependent genotoxic effect of sevofluorane determined by the CBMN assay after correction for the drug's toxicity using the *in vitro* survival curves obtained from the PNT2 cells. We identified a genotoxic effect with characteristics of a powerful *in vitro* chemical mutagen with characteristic similar to those described for γ- or X-radiations. 

After the first studies that showed increment in the frequency of MN yield, a possible genotoxic effect of sevofluorane was suggested 20, however, later studies showed contradictory results. Increment of sister chromatid exchanges (SCE) was detected in adults patients undergoing anaesthesia after 60 min exposure to the drug 21 ^,^ 22. The increment in SCE in the group exposed to the anaesthetic substances was compared with another group of medical personnel taken as a control group 22; however, increment of SCE in children subjected to the anaesthetic sevofluorane could not be established after 50 min exposure, and the increment of MN observed in these children was not statistically significant which is similar to a study described in persons occupationally exposed to the inhalation of anaesthetics gases and in patients exposed to sevofluorane 23.

Different authors argue that the results obtained by MN assay (CBMN and Comet assays) contradict with those obtained with the SCE assay^24^. In this case, it has been demonstrated that the MN assay under conditions of low level occupational exposure to sevofluorane was not associated with an increased formation of MN 24. Our study also demonstrates that a small dose of sevofluorane (5 µL) does not lead to an increment in the frequency of MN, showing a non genotoxic effect at this dose.

However, we suggest that MN tests have a limitation when it comes to evaluating the genotoxicity of sevofluorane: MN tests have very high sensitivity thresholds (detection limit), so that agents that are not intensely genotoxic are not detected. Really, the main disadvantage of the most used micronucleus assay (CBMN) is related to the variable micronucleus background frequency, so that only *in vivo* exposures in excess of 20-30 cGy X-rays can effects be detected 25. Indeed, as shown in our experiment, agents or doses like mildly toxic sevofluorane in short term exposure assays may be undetectable under numerous experimental conditions, leading to the conclusion that they have no genotoxic effect.

In a similar way, some authors have demonstrated the absence of genotoxic effect of small doses of sevofluorane using the Comet assay 23 ^,^ 24. Further, it is also worthwhile to point out that an incubation of PBL with 1% DMSO alone which is used as a solvent for anaesthetics in routine procedures was followed by measurable decrease in comet length 23. The study revealed that DNA damage by sevofluorane did not differ from the results observed for the DMSO control, hence it was concluded that small doses of sevofluorane does not exert genotoxic activity *in vitro*. The authors described that a decrease in mean comet length in PBL mediated by dissolving in DMSO by anaesthetists can be explained in two ways: (i) stabilization of the cell walls by DMSO or (ii) inhibitory influence of CYP2E isoform of cytochrome P450 responsible for the activation of sevofluorane and analogous compounds 23.

The results of our DMSO studies and that of lower doses (5 µL) of sevofluorane used in our assays are similar and do not present significant differences. DMSO is a potent antioxidant with the classic characteristics similar to potent sulphur containing radioprotectors and do offer both *in vivo* and *in vitro*, genoprotection by moderating the damage induced by the ionizing radiation. This genoprotective capacity could be attributed to their capacity to eliminate of free radicals from biological systems when present before irradiation 8 ^,^ 9. Since the effect of sevofluorane could also be due to the induction of oxidative stress, the DMSO used could help to conceal its genotoxic effect at very low dose of sevofluorane. When the doses of sevofluorane are sufficiently high, it provokes significant genotoxicity *in vivo* which compares well with response obtained by the Comet test 6 where substantial increase in the frequency of MN in peripheral blood lymphocytes was observed in all exposed groups of animals. Our results also show significant genotoxicity of sevofluorane at high dose which reaches a maximum yield of MN even with pre-toxic doses of (40 µL) which is similar to response generated by exposure to 2 Gy of X-rays.

Different studies have demonstrated that reactive oxygen species (ROS) assist in radioprotection when preconditioned with sevofluorane. Moreover, sevofluorane can also directly trigger the formation of peroxynitrite and is known to significantly increase intracellular H_2_O_2,_ superoxide anion and nitric oxide (NO) levels in PMN after 1 h of treatment thereby intensifying the depletion of intracellular glutathione (GSH) 7. Oxidative stress is the mechanism of action employed by sevofluorane to induce chromosomal damage, similar to the action of X-rays. 

Our results show that the combined treatment of sevofluorane + X-rays elicits an additive or synergistic effect and this would explain the increase in genotoxicity observed in this present study. Reactive species derived from NO inhibit enzymes, fragments DNA, modify bases, oxdidatively destroy membrane lipids, and consume cellular antioxidants, explaining the effect we have seen in the combined treatment of sevofluorane + IR. GSH, the most prominent intracellular thiol is generally regarded as a radioprotector for its ability to act as an important nucleophile in a number of detoxification reactions 9. A diminution of the intracellular levels of GSH, thereby increases the sensitivity of cells to subsequent exposure to radiation. The observation of our combined treatments using sevofluorane and IR could act in a similar way to the radiosensitization effect of cisplatin as explained previously 6.

The mechanisms of action described in oxidative stress, the formation of free radicals and a fall in the endogenous levels of antioxidants are similar to the mechanisms of action of ionizing radiation both in regards to cell death and genotoxic capacity that was previously described for exposure to X-rays 9 and γ-irradiation with radioactive caesium 8. Different authors suggest the use of antioxidants supplementation to manage /or reduce the genotoxic damage caused by waste anaesthetic gases in occupational exposure in order to reduce the genotoxic effect and oxidative stress. Similarly, others claim that the use of antioxidant substances as part of the human diet (RO) may offer protection against biological damage induced by IR in workers professionally exposed to radiation and patients undergoing radiological examinations in diagnostic radiology and nuclear medicine.

## Conclusion

Administration *in vitro* of sevofluorane at high but non-toxic doses is genotoxic to cells and show a genotoxic effect similar to that induced by 2 Gy of X-rays.
